# Power comparison between population-based case–control studies and family-based transmission–disequilibrium tests: An empirical study

**DOI:** 10.4103/0971-6866.80355

**Published:** 2011-05

**Authors:** Tanushree Haldar, Saurabh Ghosh

**Affiliations:** Human Genetics Unit, Indian Statistical Institute, Kolkata, West Bengal, India

**Keywords:** Allelic association, informative trios, complex genetic disorder

## Abstract

**BACKGROUND:**

There are two major classes of genetic association analyses: population based and family based. Population-based case–control studies have been the method of choice due to the ease of data collection. However, population stratification is one of the major limitations of case–control studies, while family-based studies are protected against stratification. In this study, we carry out extensive simulations under different disease models (both Mendelian as well as complex) to evaluate the relative powers of the two approaches in detecting association.

**MATERIALS AND METHODS:**

The power comparisons are based on a case–control design comprising 200 cases and 200 controls versus a Transmission Disequilibrium Test (TDT) or Pedigree Disequilibrium Test (PDT) design with 200 informative trios. We perform the allele-level test for case–control studies, which is based on the difference of allele frequencies at a single nucleotide polymorphism (SNP) between unrelated cases and controls. The TDT and the PDT are based on preferential allelic transmissions at a SNP from heterozygous parents to the affected offspring. We considered five disease modes of inheritance: (i) recessive with complete penetrance (ii) dominant with complete penetrance and (iii), (iv) and (v) complex diseases with varying levels of penetrances and phenocopies.

**RESULTS:**

We find that while the TDT/PDT design with 200 informative trios is in general more powerful than a case–control design with 200 cases and 200 controls (except when the heterozygosity at the marker locus is high), it may be necessary to sample a very large number of trios to obtain the requisite number of informative families.

**CONCLUSION:**

The current study provides insights into power comparisons between population-based and family-based association studies.

Association mapping of susceptible genes underlying complex disorders is an active area of current research in genetic epidemiology. Compared with Mendelian disorders, there has been limited success in identifying genes involved in complex disorders as these traits are believed to be controlled by multiple loci, some with minor gene effects, and genetic variation at any one locus does not completely determine the trait. Moreover, epistatic as well as gene–environment interactions often modify the risk of developing the disease. While linkage analyses[1] have been traditionally successful in identifying rare variants with large genetic effect sizes characterizing Mendelian disorders, they have been relatively unsuccessful in detecting common variants with moderate effect sizes characterizing complex disorders. There is evidence that association studies, which measure the extent of linkage disequilibrium (LD) between alleles of two loci,[2] are statistically more powerful than linkage studies in gene mapping of complex traits.[3] This is because LD exists over small distances on the genome, while linkage exists over larger distances. Thus, a positive association finding gives a more precise location of a locus responsible for the trait. The most popular design for genetic association studies is population-based case–control studies due to the ease of data collection and statistical methodology of testing for association. However, such studies suffer from a major inherent limitation: the problem of population stratification.[4] If the sample is a mixture of genetically heterogeneous subpopulations (i.e., there is heterogeneity in allele frequencies at the SNPs across subpopulations), the association finding may be spurious. This problem is of specific relevance for studies on Indian populations due to the increasing evidence of genetic heterogeneity among different ethnic populations in India.[5–7] While there are some statistical methods[8–10] to adjust for population stratification, it remains unclear as to the optimal number of genome-wide markers required to evaluate the level of stratification and the extent of possible correction of the relevant statistics. Thus, it has been of interest to explore, for family-based studies, alternatives that attempt to detect patterns of preferential transmission of a specific parental allele to the offspring, the most well known being the Transmission Disequilibrium Test (TDT).[11] The major advantage of this test is that it is protected against population stratification, although it requires a relatively more demanding data compared with case–control studies.

In this study, we carry out extensive simulations to compare the statistical powers of population-based case–control analyses and the family-based TDT and Pedigree Disequilibrium Test (PDT)[12] for a wide spectrum of genetic disease models. The major challenge lies in the fact that a direct and straightforward power comparison is not possible in the strict statistical sense because the study designs are different with respect to data requirements.

## Materials and Methods

We have performed the allele-level test for case–control studies, which is based on the difference of allele frequencies at a single nucleotide polymorphism (SNP) between unrelated cases and controls. The test statistic is distributed as Chi-squares with 1 d.f. under the null hypothesis of no allelic association. The TDT and the PDT are based on preferential allelic transmissions at a SNP from heterozygous parents to the affected offspring. Although the PDT has been designed to incorporate large pedigrees, we have restricted our PDT analyses to trios (two parents and an offspring) for meaningful sample size comparisons with the classical TDT. The test statistics of both TDT and PDT follow a Chi-square distribution with 1 d.f. under the null hypothesis of no linkage or no association. However, in order to compare the powers of the two designs, we need to have a consistency of the null hypotheses. We simulated the TDT/PDT design in the presence of linkage and, hence, tested only for the presence of association. The power comparisons are based on a case–control design comprising 200 cases and 200 controls versus a TDT or PDT design with 200 informative trios (i.e., having at least one parent heterozygous at the marker locus). Because, in practice, it is not possible to directly screen informative trios, we have determined the number of trios that need to be screened to obtain 200 informative trios. We have also estimated the number of cases in a case–control design with an equal number of cases and controls required to obtain equivalent power as the TDT/PDT design. We have considered five disease modes of inheritance: (i) recessive with complete penetrance (an individual is affected if and only if he/she has two copies of the disease allele), (ii) dominant with complete penetrance (an individual is affected if and only if he/she has at least one copy of the disease allele) and (iii), (iv) and (v) complex diseases with varying levels of penetrances and phenocopies (none of the risk genotypes completely determines the disease and some individuals manifest the disease in spite of not possessing any risk allele).

For the case–control design, the genotypes are generated conditioned on the disease (case/control) status under the assumption of Hardy-Weinberg genotypic proportions. For the TDT/PDT analyses, the genotypes of the parents are generated (using Hardy-Weinberg proportions) to determine whether the trio could be informative and, if so, the genotype of the offspring is generated conditioned on the parental genotypes. The affection status of the offspring is generated conditioned on the genotype at the disease locus and the family is considered for analyses only if he/she is affected. The powers are determined empirically based on 1000 replicated sets of simulated data.

## Results

The results of the power comparisons of the case–control and the TDT/PDT designs are presented in Tables 1–5, corresponding to the five disease models considered: the first, a recessive model with disease allele frequency 0.3 (prevalence of 9%); the second, a dominant model with disease allele frequency 0.05 (prevalence of 9.75%); the third, a complex disease model with a risk allele frequency 0.1 and penetrances 0.5, 0.25 and 0.05 (prevalence of 9.05%); the fourth, a complex disease model with a risk allele frequency 0.05 and penetrances 0.3, 0.15 and 0.05 (prevalence of 6.01%); the fifth, a complex disease model with a risk allele frequency 0.1 and penetrances 0.25, 0.1 and 0.05 (prevalence of 6.1%). The powers are evaluated for three marker allele frequencies: *m* = 0.1, 0.3, 0.5; two parameter values of the recombination fraction: θ= 0.05, 0.01 and four levels of LD: *D*’ = 0 (no allelic association), 0.33, 0.67, 1.0 (complete LD). Consistent with intuitive expectations, we find that the TDT/PDT design with 200 informative trios is more powerful than a case–control design with 200 cases and 200 controls, except when the heterozygosity of the marker locus is high (*m* = 0.5). However, because informative families can be ascertained only after genotyping of parents, a more appropriate comparison would be based on the number of families to be screened in a TDT/PDT design to obtain 200 informative families. It is obvious that the number of families to be screened will increase with a decrease in heterozygosity at the marker locus, and is clearly validated from all the tables, although the sample size requirement decreases faster for lower marker allele frequencies (0.1–0.3 compared with 0.3–0.5). Similarly, the number of families to be screened will decrease with an increase in the value of *D*’. This follows from the fact that the sample size requirement depends on the frequencies of the haplotypes based on the marker locus and the disease locus. However, it is interesting to note that although the power of the TDT/PDT is higher for smaller values of θ, the number of families to be screened does not depend on θ. Irrespective of the disease model, we find that for equivalent power, the number of families to be screened in a TDT/PDT design far outnumber the number of cases in a case–control design with equal number of cases and controls. The difference in the sample size requirements becomes less pronounced with (i) increase in heterozygosity, (ii) decrease in the value of *D*’ and (iii) decrease in the penetrance values (i.e., with an increasing degree of disease complexity). In order to evaluate the effect of sample size on the relative powers of the two designs, we simulated data on *N* cases and *N* controls as well as *N* informative families under the five models for *N* = 100, 500. Although the results are not presented for brevity, the qualitative inferences are similar to those described above. However, we observe that with an increase in N, the difference in the powers between the two designs becomes negligible, although the number of families that need to be screened to get a larger number of informative families would also increase.

**Table 1:** Power comparisons under a recessive model with disease allele frequency 0.3 (prevalence of 9%)

				Power			
*m*	D	θ	CC	TDT	PDT	*N1*	*N2*
	0	0.05	0.050	0.043	0.046	611.38	
		0.01		0.050	0.052	610.42	
	0.33	0.05	0.957	0.996	0.997	470.11	280
0.1		0.01		>0.999	0.998	468.96	320
	0.67	0.05	>0.999	>0.999	>0.999	386.68	*
		0.01		>0.999	>0.999	387.54	*
	1	0.05	>0.999	>0.999	>0.999	333.53	*
		0.01		>0.999	>0.999	334.27	*
	0	0.05	0.055	0.050	0.051	301.52	
		0.01		0.040	0.039	301.83	
	0.33	0.05	>0.999	>0.999	>0.999	261.66	*
0.3		0.01		>0.999	>0.999	261.76	*
	0.67	0.05	>0.999	>0.999	>0.999	236.83	*
		0.01		>0.999	>0.999	236.63	*
	1	0.05	>0.999	>0.999	>0.999	219.92	*
		0.01		>0.999	>0.999	219.60	*
	0	0.05	0.060	0.046	0.043	266.99	
		0.01		0.048	0.048	266.51	
	0.33	0.05	0.999	>0.999	>0.999	266.24	*
0.5		0.01		>0.999	>0.999	267.19	*
	0.67	0.05	>0.999	>0.999	>0.999	266.4	*
		0.01		>0.999	>0.999	266.89	*
	1	0.05	>0.999	>0.999	>0.999	266.79	*
		0.01		>0.999	>0.999	266.93	*

CC = case control; *N1*, number of families to be screened to obtain 200 informative families; *N2*, number of cases/controls required to get power equivalent to TDT/PDT with 200 informative families;

* CC = case control; *N1*, number of families to be screened to obtain 200 informative families; *N2*, number of cases/controls required to get power equivalent to TDT/PDT with 200 informative families;

**Table 2:** Power comparisons under a dominant model with disease allele frequency 0.3 (prevalence of 9.75%)

				Power			
*m*	D’	θ	CC	TDT	PDT	*N1*	*N2*
0.1	0	0.05	0.048	0.046	0.049	612.32	
		0.01		0.050	0.049	611.13	
	0.33	0.05	>0.999	>0.999	>0.999	383.92	*
		0.01		>0.999	>0.999	383.89	*
	0.67	0.05	>0.999	>0.999	>0.999	279.28	*
		0.01		>0.999	>0.999	278.63	*
	1	0.05	>0.999	>0.999	>0.999	218.34	*
		0.01		>0.999	>0.999	218.04	*
0.3	0	0.05	0.06	0.051	0.054	301.33	
		0.01		0.050	0.045	301.17	
	0.33	0.05	0.972	0.950	0.944	279.16	*
		0.01		0.974	0.974	278.94	*
	0.67	0.05	>0.999	>0.999	>0.999	259.57	*
		0.01		>0.999	>0.999	259.84	*
	1	0.05	>0.999	>0.999	>0.999	242.85	*
		0.01		>0.999	>0.999	242.48	*
0.5	0	0.05	0.06	0.060	0.055	266.72	
		0.01		0.050	0.047	266.96	
	0.33	0.05	0.709	0.650	0.658	266.21	*	
		0.01		0.752	0.752	267.14	215	
	0.67	0.05	0.998	0.999	0.999	266.42	*	
		0.01		>0.999	>0.999	267.06	*
	1	0.05	>0.999	>0.999	>0.999	266.43	*	
		0.01		>0.999	>0.999	266.04	*

**Table 3:** Power comparisons under a complex disease model with a risk allele frequency 0.1 and penetrances 0.5, 0.25 and 0.05 (prevalence of 9.05%)

				Power			
*m*	D’	θ	CC	TDT	PDT	**N1**	**N2**
0.1	0	0.05	0.048	0.042	0.044	609.64	
		0.01		0.060	0.059	610.00	
	0.33	0.05	0.896	0.972	0.976	480.31	300
		0.01		0.990	0.992	479.53	380
	0.67	0.05	>0.999	>0.999	>0.999	396.79	*
		0.01		>0.999	>0.999	397.51	*
	1	0.05	>0.999	>0.999	>0.999	337.45	*
		0.01		>0.999	>0.999	337.91	*
0.3	0	0.05	0.060	0.050	0.049	302.57	
		0.01		0.060	0.055	301.37	
	0.33	0.05	0.413	0.380	0.374	290.98	*
		0.01		0.470	0.477	290.56	230
	0.67	0.05	0.949	0.930	0.931	280.47	*
		0.01		0.950	0.952	280.84	*
	1	0.05	0.999	>0.999	>0.999	271.66	*
		0.01		>0.999	>0.999	271.60	*
0.5	0	0.05	0.060	0.060	0.052	266.62	
		0.01		0.060	0.058	266.62	
	0.33	0.05	0.219	0.180	0.181	266.87	*
		0.01		0.220	0.223	266.97	*
	0.67	0.05	0.659	0.630	0.626	266.63	*
		0.01		0.670	0.677	266.29	*
	1	0.05	0.946	0.914	0.916	266.57	*
		0.01		0.960	0.957	266.56	215

**Table 4:** Power comparisons under a complex disease model with a risk allele frequency 0.05 and penetrances 0.3, 0.15 and 0.05 (prevalence of 6.01%)

				Power			
*m*	D’	θ	CC	TDT	PDT	*N1*	*N2*
0.1	0	0.05	0.050	0.040	0.043	610.58	
		0.01		0.050	0.050	610.01	
	0.33	0.05	0.225	0.400	0.398	552.83	400
		0.01		0.490	0.494	555.36	520
	0.67	0.05	0.659	0.890	0.892	506.24	350
		0.01		0.940	0.939	507.82	460
	1	0.05	0.918	0.990	0.995	468.84	300
		0.01		0.997	0.998	468.44	380
0.3	0	0.05	0.060	0.060	0.052	301.53	
		0.01		0.050	0.054	301.63	
	0.33	0.05	0.094	0.100	0.101	297.31	275
		0.01		0.100	0.095	297.13	275
	0.67	0.05	0.247	0.260	0.259	293.05	205
		0.01		0.320	0.319	293.02	275
	1	0.05	0.477	0.485	0.488	288.96	210
		0.01		0.590	0.589	289.52	260
0.5	0	0.05	0.060	0.060	0.058	267.03	
		0.01		0.050	0.047	266.11	
	0.33	0.05	0.073	0.070	0.073	266.82	*
		0.01		0.080	0.085	266.75	295
	0.67	0.05	0.175	0.130	0.121	266.46	*
		0.01		0.160	0.154	266.71	*
	1	0.05	0.259	0.230	0.230	266.80	*
		0.01		0.280	0.277	266.43	240

**Table 5:** Power comparisons under a complex disease model with a risk allele frequency 0.05 and penetrances 0.25, 0.1 and 0.05 (prevalence of 6.1%)

				Power			
*m*	D’	θ	CC	TDT	PDT	*N1*	*N2*
0.1	0	0.05	0.050	0.051	0.051	610.72	
		0.01		0.042	0.045	610.70	
	0.33	0.05	0.290	0.445	0.449	548.41	350
		0.01		0.570	0.569	549.10	480
	0.67	0.05	0.769	0.958	0.954	498.3	400
		0.01		0.980	0.980	500.35	450
	1	0.05	0.970	0.998	0.998	459.66	320
		0.01		1.000	1.000	460.13	330
0.3	0	0.05	0.060	0.062	0.063	301.98	
		0.01		0.050	0.050	301.36	
	0.33	0.05	0.112	0.118	0.125	296.65	215
		0.01		0.130	0.133	296.11	220
	0.67	0.05	0.318	0.327	0.339	292.22	215
		0.01		0.390	0.383	292.07	270
	1	0.05	0.583	0.622	0.619	288.82	210
		0.01		0.690	0.689	287.55	255
0.5	0	0.05	0.060	0.050	0.049	266.56	
		0.01		0.050	0.045	266.78	
	0.33	0.05	0.080	0.066	0.062	266.87	*
		0.01		0.070	0.063	266.43	*
	0.67	0.05	0.211	0.177	0.174	266.52	*
		0.01		0.190	0.191	266.57	*
	1	0.05	0.350	0.320	0.314	266.06	*
		0.01		0.344	0.336	266.52	*

## Discussion

It is clear from the analytical studies that population-based genetic case–control designs suffer from the inherent limitation of population stratification. Because it is infeasible in most scenarios to ascertain whether the samples collected for case–control association analyses are genetically homogeneous, novel positive findings are always susceptible to be false-positives. The family-based tests for association such as the TDT and PDT circumvent the problem of population stratification as positive association findings based on these tests are possible only in the presence of linkage between the marker locus and the disease locus. However, the data requirements (such as informative trios) for family-based association analyses are much more demanding compared with case–control studies. Thus, there are both advantages and limitations of the two study designs. In this light, the current study provides an alternative framework for statistical comparison based on power.

We found that while the TDT or PDT based on a set of informative trios is more powerful in detecting association compared with a case–control design comprising an equal number of cases and controls as the number of informative trios except when the heterozygosity of the marker locus is very high, a more fair statistical comparison of the total number of trios screened in the TDT or PDT analysis with the number of cases (or controls) in a case–control design to obtain equivalent power shows that the case–control design wins the battle of sample sizes very comprehensively. Moreover, it needs to be emphasized that while a case–control design comprising *N* cases and *N* controls requires genotyping of *2N* individuals, a TDT or PDT design with *N* trios requires an expected genotyping of (2+α) N individuals, where α is the proportion of informative trios. Thus, in view of the fact that the case–control design yields more power than the TDT/PDT where the number of cases (or controls) equals the number of trios, the relative gain in a case–control design is even greater when the genotyping costs are taken into consideration. We would like to highlight that while family-based association analyses are protected against population stratification with respect to false-positives, they may be adversely affected with respect to false-negatives. Thus, it is of interest to compare the powers of the case–control design to the TDT/PDT in the presence of population stratification. This is statistically challenging as population stratification induces an inflated rate of false-positives in the case–control framework and, hence, a direct comparison of powers without adjusting the distributional thresholds for stratification is not statistically valid.

We plan to carry out extensive simulations under population stratification and compare the powers of the two procedures after adjustments of stratification in the case–control analyses based on a principal components approach.[10]
